# Novel heterozygous *GATA3* and *SLC34A3* variants in a 6‐year‐old boy with Barakat syndrome and hypercalciuria

**DOI:** 10.1002/mgg3.1222

**Published:** 2020-03-10

**Authors:** Sha Yu, Wen‐xia Chen, Wei Lu, Chao Chen, Yihua Ni, Bo Duan, Bin Wang, Huijun Wang, Zheng‐min Xu

**Affiliations:** ^1^ Department of Otolaryngology‐Head and Neck Surgery Children’s Hospital of Fudan University Shanghai China; ^2^ Center for Molecular Medicine Pediatrics Research Institute Children’s Hospital of Fudan University Shanghai China; ^3^ Endocrinology and Inherited Metabolic Diseases Children’s Hospital of Fudan University Shanghai China

**Keywords:** Barakat syndrome, *GATA3*, hypercalciuria, sensorineural deafness, *SLC34A3*

## Abstract

**Background:**

Barakat syndrome is an autosomal dominant disorder characterized by the triad of hypoparathyroidism, sensorineural deafness, and renal anomalies and is caused by mutations in *GATA3* gene. *SLC34A3* is the cause gene of hypophosphatemic rickets with hypercalciuria, and heterozygous carriers may have milder clinical symptoms. The aim of this study was to identify the underlying genetic cause of a patient who initially presented with renal failure, hypercalciuria, kidney stone, and bilateral sensorineural deafness.

**Methods:**

A 6‐year‐old boy with complex clinical presentations was investigated. Comprehensive medical evaluations were performed including auditory function tests, endocrine function tests, metabolic studies, and imaging examinations. Molecular diagnoses were analyzed by trio whole‐exome sequencing.

**Results:**

One novel de novo deleterious variant (c. 324del) of the *GATA3* gene was identified in the patient. The patient can be diagnosed with Barakat syndrome. In addition, one novel variant (c. 589A>G) of the *SLC34A3* gene was detected, which was inherited from the father. This heterozygous variant can explain the hypercalciuria and kidney stone that occurred in both the patient and his father.

**Conclusion:**

This study provides a special case which is phenotype‐driven dual diagnoses, and the two novel variants can parsimoniously explain the complex clinical presentations of this patient.

## Graphical abstract

A 6‐year‐old boy with Barakat syndrome and early‐onset hypercalciuria. One novel de novo variant in *GATA3*, and one novel variant in *SLC34A3* inherited from symptomatic father.

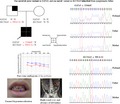

## 1 INTRODUCTION

Barakat syndrome, also known as hypoparathyroidism‐deafness‐renal anomalies (HDR) syndrome [MIM:146255] was first reported in 1977 as a rare autosomal dominant disorder characterized by the triad of hypoparathyroidism (H), sensorineural deafness (D), and renal anomalies (R) (Barakat, D’Albora, Martin, & Jose, 1977; Hasegawa et al., 1997). The exact prevalence of the disease is unclear. GATA‐binding protein 3 (GATA3, encoded by *GATA3* gene) is a dual‐zinc finger transcription factor, located on chromosome 10p14, which is essential in the normal development of many fetal tissues, including inner ear, breast, parathyroid glands, and kidney tissue (Grigorieva et al., 2010; Grote, Souabni, Busslinger, & Bouchard, 2006; Van Esch et al., 2000). Furthermore, GATA3 is required for cell differentiation and maturation in postnatal development, such as parathyroid chief cells, inner hair cells, and Th2 lymphocytes (Bardhan et al., 2019; Grigorieva et al., 2010; Hosoya, Maillard, & Engel, 2010). *GATA3* haploinsufficiency due to loss‐of‐function mutations or deletions is the known pathogenic mechanism causing HDR syndrome (Bardhan et al., 2019; Van Esch et al., 2000).

Homozygous or compound heterozygous mutations involving *SLC34A3* have been associated with hereditary hypophosphatemic rickets with hypercalciuria (HHRH, MIM: 241530; Mejia‐Gaviria, Gil‐Peña, Coto, Pérez‐Menéndez, & Santos, 2010). Most recently, heterozygous mutations of the *SLC34A3* gene have been identified in individuals with milder clinical symptoms, including isolated hypercalciuria, mild hypophosphatemia, and elevated 1,25‐dihydroxy vitamin D [1,25‐(OH) 2 D] (Dasgupta et al., 2014).

Here, we report a 6‐year‐old boy with the triad of HDR syndrome, enamel hypomineralization, and hypercalciuria. The latter cannot be explained by HDR syndrome due to the inappropriate onset time; the complex clinical presentation is likely due to a de novo frameshift variant in *GATA3* and a missense variant in *SLC34A3*.

## 2 MATERIALS AND METHODS

### 2.1 Patient

The proband was evaluated in a clinical setting by multiple specialists, including otolaryngologists, endocrinologists, and geneticists. Pure tone audiometry (PTA) was recorded using an Interacoustics AC40 clinical audiometer and HAD‐280 earphones. Speech audiometry was performed with standard Chinese single syllable word chart cards. The audiological classifications are shown in Appendix S1.

The genetic testing was approved by the ethics committees of Children's Hospital, Fudan University (2014‐107 and 2015‐130). Informed consent of clinical information and photographs were obtained from patients’ parents.

### 2.2 Genetic studies

See Appendix S1.

## 3 RESULTS

### 3.1 Clinical characteristics

The patient, a 6‐year‐old boy, was the first child of nonconsanguineous Chinese parents with no history of renal hypo/dysplasia, deafness, or hypoparathyroidism. After a normal pregnancy, he was born by spontaneous vaginal delivery at 38 weeks of gestation. Apgar scores were 10/1 and 10/5. Growth parameters showed birth weight of 3,200 g (in the 50th–70th percentile), head circumference of 34.5 cm (50th percentile), and length of 51 cm (in the 50th–70th percentile). In the newborn period, he failed the newborn hearing screening, which was detected by transiently evoked otoacoustic emissions. However, subsequent hearing evaluations were not made.

**Figure 1:**
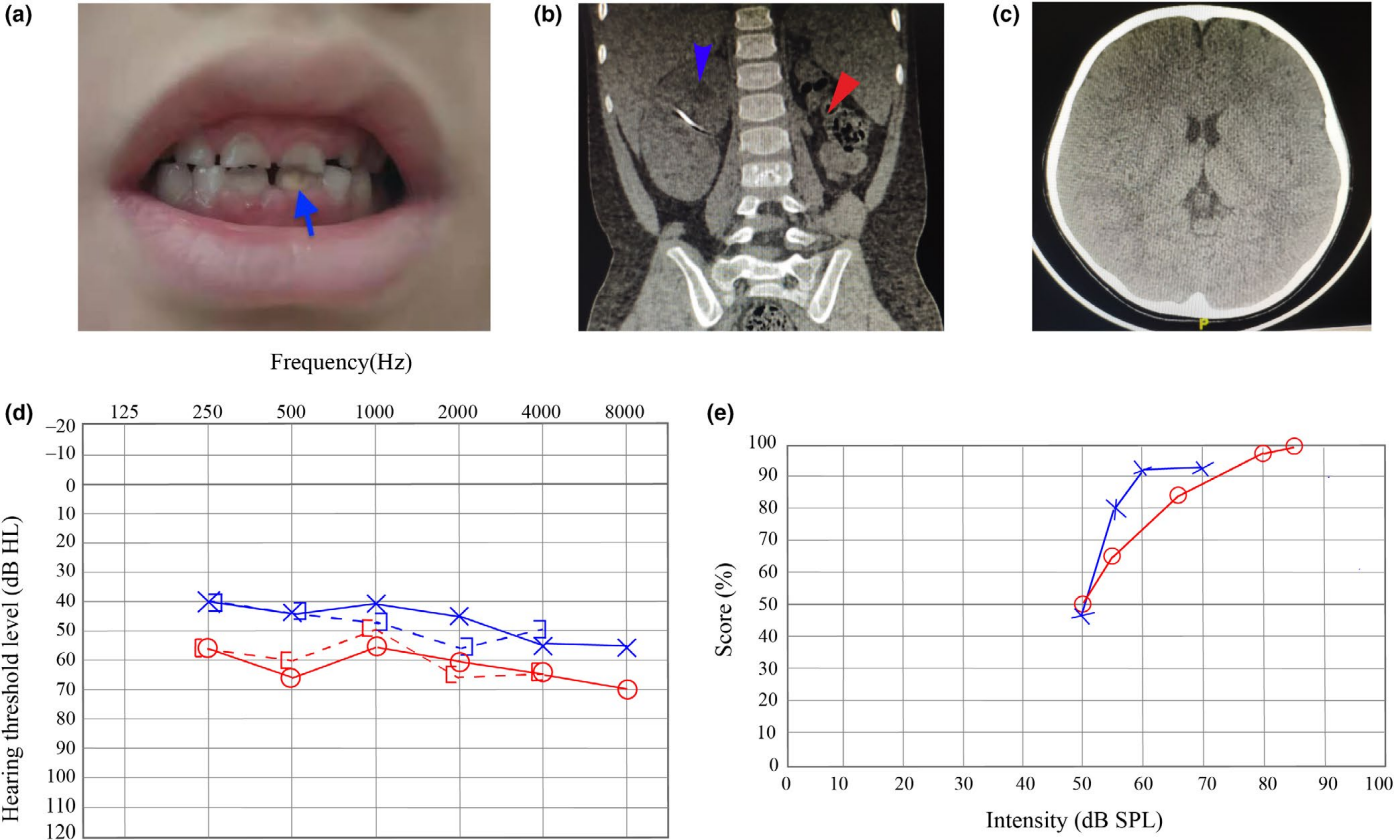
Clinical characteristics of the patient. (a) Enamel hypomineralization on the permanent incisors (blue arrow). (b) Abdominal computed tomography (CT) showing renal cyst of right kidney (blue arrow) and absence of left kidney (red arrow). (c) Normal brain CT (d) Pure‐tone audiogram of the proband. Red and blue indicate thresholds for the right and left ears, respectively. Solid and dashed lines refer to air‐ and bone‐conduction, respectively. Bilateral flat moderate to moderately severe sensorineural deafness is observed. (e) Speech audiometry. Red and blue indicate speech recognition for the right and left ears, respectively. The speech recognition threshold is approximately 50 dB in both ears

At 5 years and 7 months of age, he was first referred to the clinical service with anuria, vomiting, and low back pain. Laboratory tests revealed acute renal failure with serum creatinine levels of 733 µmmol/L and urea levels of 28.5 mmol/L. Abdominal ultrasound examination and computed tomography (CT) showed ureteral calculi and renal cyst of right kidney, and absence of left kidney (Figure 1). Then, he received the treatment of hemodialysis and extracorporeal shock wave lithotripsy. Analysis of his stone composition obtained from the surgery showed calcium oxalate stones.

At 6 years of age, he had hearing evaluations and laboratory tests due to the results of genetic testing. DPOAEs were absent bilaterally. PTA revealed bilateral flat audiogram configurations with moderate sensorineural deafness for the left ear and moderately severe sensorineural deafness for the right ear (Figure 1). Speech audiometry showed that the speech recognition threshold was approximately 50 dB in both ears. Moreover, 100% speech recognition was attained at 85 dB sound level in the right ear, whereas in the left ear a maximum of 92% speech recognition was attained at the 60 dB sound level (Figure 1). Speech in the noise test showed that 70% speech recognition was attained at the signal‐to‐noise of 5 dB with a 70‐dB sound level, and 0% speech recognition was attained at the signal‐to‐noise of –5 dB. Subsequently, he was fitted with hearing aids.

Laboratory testing at the age of 6 years revealed hypocalcemia with a serum calcium level of 2.1 mmol/L (normal, 2.2–2.65 mmol/L), hyperphosphatemia with a serum phosphorus level of 12.34 mmol/L (normal, 1.0–1.95 mmol/L), hypoparathyroidism with a serum intact parathyroid hormone (PTH) level of 0.7 pmol/L (normal, 1.06–7.31 pmol/L), hypercalciuria with a 24‐hr urinary calcium level of 53.67 mg (normal, 1.96–2.66 mg), and slightly reduced serum 1,25(OH)_2_D 20 ng/ml (normal, 21–65 ng/ml). Other biochemical studies were normal, including serum (25(OH)D), serum creatinine, urine creatinine, and alkaline phosphatase. In addition, immunological analyses were normal, including immunoglobulin (Ig)G, IgM, and IgA concentrations, T cell subsets, and T cell and B cell counts. Thereafter, treatment was initiated with oral calcium and calcitriol (25 µg, qod) supplementation, which restored normal blood calcium.

At this stage, he was diagnosed with enamel hypomineralization. He had tooth pain and difficulties in tooth brushing. Tooth examination showed the permanent lower first molars and incisors had distinct opacities, and the primary teeth had caries (Figure 1). At the recent follow‐up (6 years and 4 months), he had his permanent upper first molars, and they were normal.

By the age of 6 years, his growth was age‐appropriate, and he had no dysmorphic features and no clinical symptoms of hypocalcemia. A CT scan of the brain (Figure 1) and the temporal bones of the left and right ears at the age of 6 demonstrated to be normal. Bone mineral density (BMD) elevated at age 6 years showed normal. Electrocardiograph was also normal.

The father was diagnosed with multiple bilateral kidney stones from the age of 25 years. Laboratory testing at the age of 30 revealed hypercalciuria with a 24‐hr urinary calcium level of 714.4 mg/24 hr (normal, <300 mg/24 hr) and elevated serum 1,25(OH)_2_D 78 ng/ml (normal, 21–65 ng/ml). The examination of PTH, 25(OH)D, serum inorganic phosphorus, serum calcium, and BMD showed normal.

### 3.2 Molecular analyses

**Figure 2:**
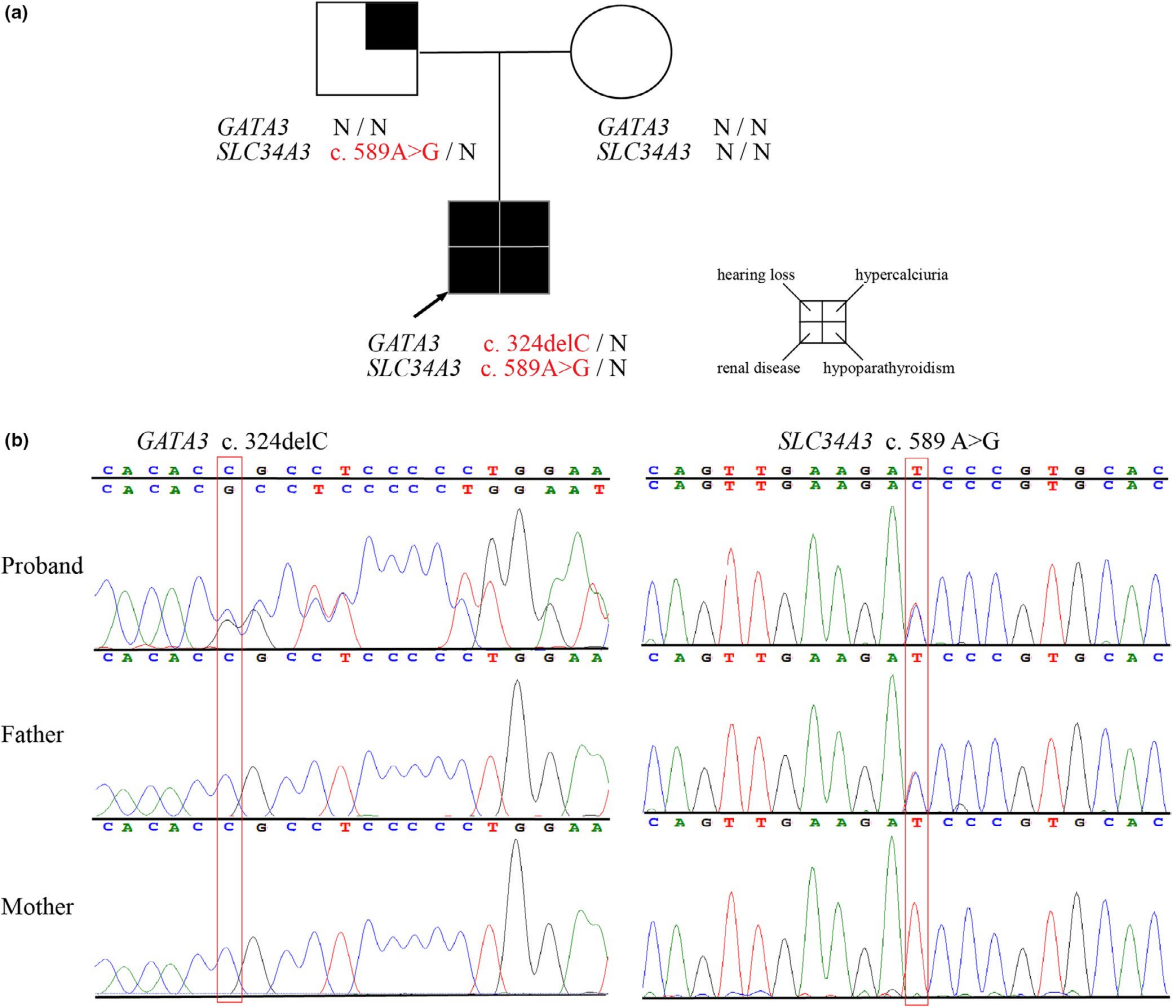
Co‐occurrence of *GATA3* and *SLC34A3* mutations (a) Pedigree. The presence of symptoms of HDR syndrome and hypercalciuria is indicated by blackening of each quartered area. The words in red indicate variants of *GATA3* or *SLC34A3*. N, wild‐type. (b) Sanger sequencing, presence of the mutated alleles is indicated by red frames

A trio WES analysis in the proband showed a de novo heterozygous frameshift variant (NM_001002295.2: c.324delC, p. Ala109Profs*86) in *GATA3* and a heterozygous missense variant (NM_001177316.2: c.589A>G, p. Ile197Val) in *SLC34A3* inherited from the father. Both variants were independently confirmed using Sanger sequencing (Figure 2).

**Figure 3:**
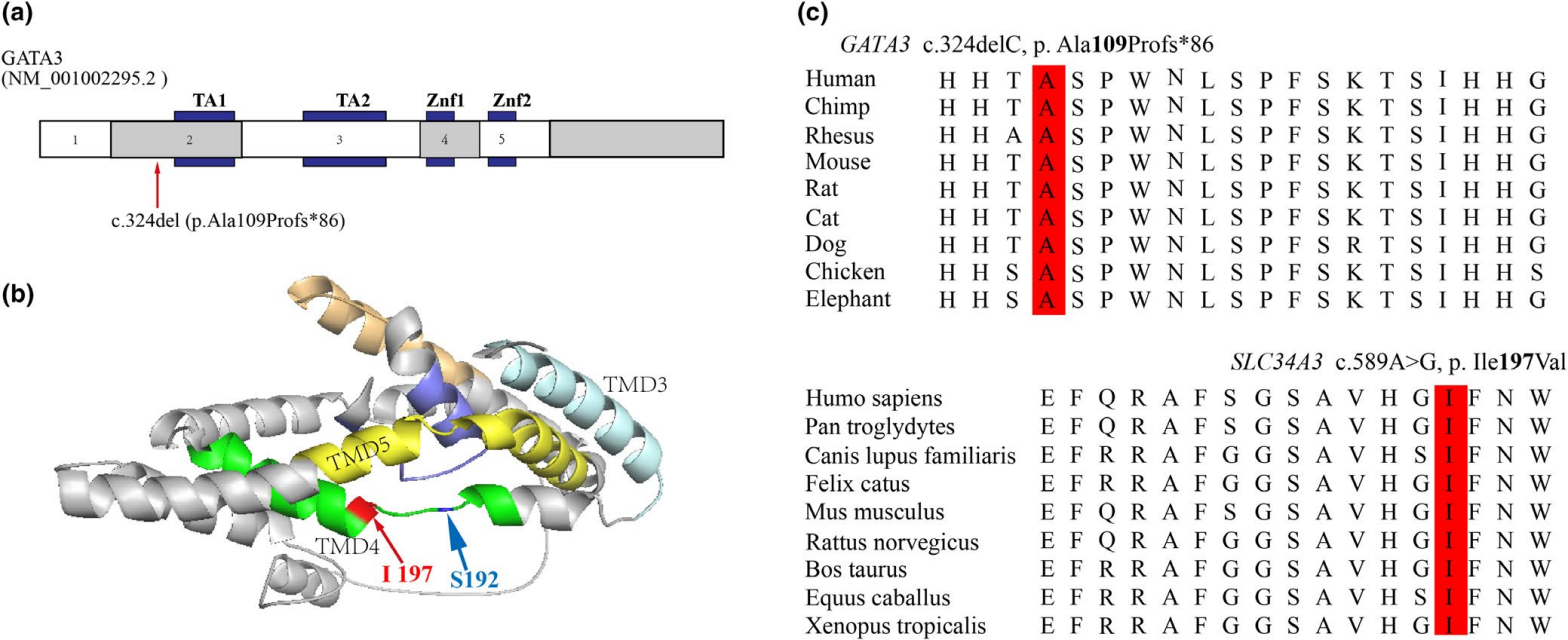
Genetic characteristics of *GATA3* and *SLC34A3* mutations. (a) Schematic representation of the *GATA3* gene, exons, and its functional domains. The location of the variant is depicted by arrows. TA1 and TA2 denote two transactivation domains, and ZF1 and ZF2 represent two zinc finger domains. (b) Model of flNaPi2c encoded by *SLC34A3* (match ID Q8N130:1) from DomSerf (http://www.genome3d.eu/uniprot/id/Q8N130/annotations), showing p. Ile197Val (red) and p. Ser192Leu (blue) localization within the fourth transmembrane (TM) (green). (c) Alignment of *GATA3* and *SLC34A3* orthologs. Amino acids in the mutation site are conserved from human to mouse and are highlighted in red

The frameshift variant (c. 324delC, p. Ala109Profs*86) in *GATA3* is absent in the ExAC (v1.0), 1,000 Genomes, and gnomAD (v2.1.1) databases. Ala109 represents a highly conserved amino acid, located before the TA1 domains. This variant is predicted to cause a premature stop codon before the zinc‐finger transcription factor 1 motif and zinc‐finger transcription factor 2 motif domains of GATA3, which is crucial for DNA binding (Figure 3). The variant is predicted to disease causing by the silico tool of Mutation Taster. According to the American College of Medical Genetics (ACMG) variant classification, it met several criteria (PVS1, PS2, and PM2) and was classified as a pathogenic variant.

The missense variant (c.589A>G, p. Ile197Val) in *SLC34A3* is absent in the ExAC, 1,000 Genomes, and gnomAD databases. The Ile197 residue is highly conserved across difference species and located within the fourth transmembrane domain (Figure 3). This variant c.589A>G is predicted to be neutral, benign, and probably harmless by silico tools from SIFT, PolyPhen‐2, and Mutation Taster, respectively. According to the ACMG variant classification, it met the criteria of PM2, and was classified as of uncertain significance. However, it was detected in three children whose data were collected in the in‐house database. The variant of the patient was inherited from the father, who also has hypercalciuria. Moreover, one of the three children, a 1‐year‐old girl, had mild hypercalciuria but no kidney stone was detected.

## 4 DISCUSSION

In this report, we describe a unique 6‐year‐old boy having combined features of HDR syndrome and hypercalciuria. Genetic testing identified two novel variants in *GATA3* and *SLC34A3* genes. Germline *GATA3* mutation is the major cause of HDR syndrome. To date, 180 cases have been reported in the literature (Barakat, Raygada, & Rennert, 2018). The frameshift variant (c. 324delC, p. Ala109Profs*86) of the *GATA3* gene found in our patient was the most common phenotype, and was expected to cause a loss of DNA binding and lead to haploinsufficiency. The disruption of the wild‐type protein could affect the proliferation and differentiation of the inner ear, parathyroid glands, and kidney (Ali et al., 2006; Barakat et al., 2018). Therefore, all the phenotypes presented in our patient (including sensorineural deafness, renal anomalies hypoparathyroidism, hypocalcemia, and hyperphosphatemia induced by hypoparathyroidism), could be explained by *GATA3* defect except for the hypercalciuria.

Hypercalciuria has been reported in patients with HDR syndrome, whereas it commonly occurs after over‐treatment with calcium and calcitriol (Kim et al., 2015). It is thought that the hypercalciuria may be caused by intestinal hyperabsorption of calcium induced by vitamin D intoxication and decreased reabsorption of tubular calcium induced by low levels of PTH (Chenouard et al., 2013). Unexpectedly, the proband in our study originally presented with hypercalciuria and ureteral calculi when the administration of calcium and vitamin D had not been started. Therefore, the hypercalciuria was not the result of vitamin D intoxication.

It is well known that various diseases or situations, such as idiopathic hypercalciuria, primary hyperparathyroidism, medullary sponge kidney, and renal tubular acidosis, can cause hypercalciuria (Favus, 2000). Considering that the proband's father also had hypercalciuria with normocalcemia, a familial form of dominant idiopathic hypercalciuria, such as dominant absorptive hypercalciuria was suspected. Dominant absorptive hypercalciuria [MIM:143870] is characterized by intestinal hyperabsorption of calcium in the presence of normocalcemia and normal immunoreactive PTH, and may be caused by mutations in the *ADCY10* gene. The majority of patients with this type of hypercalciuria had idiopathic hypercalciuria, calcium oxalate nephrolithiasis, and low spinal bone density (Akbari et al., 2019; Reed et al., 2002). However, no rare or pathogenic variation was found in the *ADCY10* gene by reanalysis of the trio‐WES data in this family, and no low spinal bone density were observed by BMD test. Hence, it is unlikely that this patient had dominant absorptive hypercalciuria.

A significant involvement of heterozygous *SLC34A3* variants has been described in patients with idiopathic hypercalciuria (Dasgupta et al., 2014; Hasani‐Ranjbar et al., 2018; Schönauer et al., 2019). This finding indicates that the heterozygous *SLC34A3* variant may be the cause of an autosomal dominant idiopathic hypercalciuria, and it may be caused by intestinal hyperabsorption of calcium and increased serum 1,25‐dihydroxyvitamin D (Dasgupta et al., 2014). Interestingly, a heterozygous variant of *SLC34A3* (c.589A>G) was found in the patient and his symptomatic father by the reanalysis of the trio WES data, especially for the causes of idiopathic hypercalciuria, including *CLCN5*,* CASR*,* CLDN16*,* CLDN19*,* ADCY10*,* SLC9A3R1*,* GLUT2*,* HSPG2*,* FN1*,* CYP24A1*,* SLC34A1*, and *SLC34A3* (Dasgupta et al., 2014; Stechman, Loh, & Thakker, 2009).

Several lines of evidence indicate that the missense variant (c.589A>G) is a disease‐causing mutation. First, this variant was not found in control individuals, including the ExAC, 1,000 Genomes, and gnomAD databases. Second, the affected residue is highly conserved during evolution and located within the fourth transmembrane domain. Moreover, a missense variant (c.575C>T, p. Ser192Leu) located within the same domain was reported to be pathogenic (Figure 3; Schönauer et al., 2019). Third, this variant co‐segregated with idiopathic hypercalciuria in the patient's father and one child of the in‐house database. In view of these points, we speculated that the heterozygous variant of *SLC34A3* may be the cause for the hypercalciuria in this family. In addition, the slightly decreased 1,25‐(OH)_2_D of the proband which should be lower due to the hypoparathyroidism caused by the mutation of the *GATA3* gene, and the increased 1,25‐(OH)_2_D of his father, reinforce our hypothesis.

Coincidently, Marina *et al*. reported that one patient with HDR syndrome presented with hypercalciuria (Häusler, 2019). Due to the limited information in this meeting abstract, no cause was found for the cause of hypercalciuria. If the hypercalciuria occurred before treatment, it is possible that *SLC34A3* mutation caused hypercalciuria, with the simultaneously found hypophosphatemia and hypercalciuria. However, without thoroughly sequencing supporting data, this is only a speculation. Therefore, our report reaffirmed the importance of trio whole‐exome sequencing and consideration of other genetic causes for patients with complex clinical phenotypes. Without whole‐exome sequencing, direct sequencing of *GATA3* would have missed the *SLC34A3* variant.

Deafness is the most consistent feature of the HDR syndrome, with 96.7% of the patients presenting with it (Barakat et al., 2018). In this study, the proband presented with moderate to moderately severe sensorineural deafness, which was bilateral, symmetric, and slightly worse at the higher end of the frequency spectrum, which has been reported previously (Barakat et al., 2018). The speech audiometry corresponded well with PTA, but he performed poorly on speech in the noise test. The latter finding and absence of DPOAE support previous studies that have demonstrated that outer hair cells play an important role in the etiology of sensorineural deafness (Bardhan et al., 2019; Chien et al., 2014). Furthermore, as shown in Table 1, no significant difference was found in the type, degree, prognosis, and configuration of deafness in patients with different *GATA3* variants, except for the profound deafness and diagnostic age. Patients with *GATA3* deletion have a higher incidence of profound deafness (*p* < .05), and this can be interpreted by the deletion of other involved genes (Melis et al., 2012).

**Table 1:** The comparison of audiological characteristics among different *GATA3* mutation types in literature

	Missense (*n* = 8)	Splicing (*n* = 5)	Frameshift (*n* = 27)	Nonsense (*n* = 2)	Deletion (*n* = 11)	Total (*n* = 53)	*p*‐Value^a^
Gender							
Male	4/8 (50%)	2/5 (40%)	8/27 (27%)	1/2 (50%)	7/11 (64%)	22/53 (42%)	NS
Female	4/8 (50%)	3/5 (60%)	19/27 (73%)	1/2 (50%)	4/11 (36%)	31/53 (58%)	NS
Age at diagnose of D							
<6 months	0/8 (0%)	1/5 (20%)	1/27 (3%)	NA	2/8 (25%)	4/48 (8%)	NS
<1 year	1/8 (13%)	4/5 (80%)	2/27 (7%)	1/2 (50%)	3/8 (38%)	11/50 (18%)	<.05*
>19 years	1/8 (13%)	0/5 (0%)	2/27 (7%)	0/2 (0%)	0/8 (0%)	3/50 (6%)	NS
Childhood	7/8 (88%)	5/5 (100%)	25/27 (93%)	2/2 (100%)	8/8 (100%)	47/50 (94%)	NS
Type of D							
Conductive D	0/8 (0%)	0/5 (0%)	0/27 (0%)	0/2 (0%)	0/11 (0%)	0/53 (0%)	NS
Sensorineural D	8/8 (100%)	5/5 (100%)	26/27 (97%)	2/2 (100%)	11/11 (100%)	52/53 (98%)	NS
Mixed D	0/8 (0%)	0/5 (0%)	1/27 (4%)	0/2 (0%)	0/11 (0%)	1/53 (2%)	NS
Degree of D							
Mild	1/8 (13%)	0/5 (0%)	3/27 (11%)	0/2 (0%)	1/11 (9%)	5/53 (9%)	NS
Moderate	3/8 (38%)	3/5 (60%)	14/27 (52%)	2/2 (100%)	4/11 (36%)	26/53 (49%)	NS
Moderately severe	4/8 (50%)	1/5 (20%)	6/27 (22%)	0/2 (0%)	1/11 (9%)	12/53 (23%)	NS
Severe	0/8 (0%)	1/5 (20%)	4/27 (15%)	0/2 (0%)	2/11 (18%)	7/53 (13%)	NS
Profound	0/8 (0%)	0/5 (0%)	0/27 (0%)	0/2 (0%)	3/11 (27%)	3/53(6%)	<.05*
Configuration of D							
Flat	0/1 (0%)	NA	13/19 (68%)	2/2 (100%)	0/2 (0%)	15/24 (63%)	NS
High‐frequency D	1/1 (100%)	NA	5/19 (26%)	0/2 (0%)	2/2 (100%)	8/24 (33%)	NS
Gently sloping	0/1 (0%)	NA	5/19 (26%)	0/2 (0%)	1/2 (50%)	6/24 (25%)	NS
Steeply sloping	0/1 (0%)	NA	0/19 (0%)	0/2 (0%)	1/2 (50%)	1/24 (4%)	NS
U‐shaped	0/1 (0%)	NA	0/19 (5%)	0/2 (0%)	0/2 (0%)	0/24 (0%)	NS
Rising	0/1 (0%)	NA	1/19 (5%)	0/2 (0%)	0/2 (0%)	1/24 (4%)	NS
Other							
Bilateral D	8/8 (100%)	5/5 (100%)	27/27 (100%)	2/2 (100%)	11/11 (100%)	53/53 (100%)	NS
Symmetrical D	1/2 (50%)	NA	19/19 (100%)	2/2 (100%)	3/3 (100%)	25/26 (96%)	NS
Progressive D	1/1 (100%)	NA	2/10 (20%)	0/2 (0%)	1/4 (25%)	4/17 (23%)	NS

Note

Detail data of these 53 patients in literature are available in Table S1.

Abbreviations: D, deafness; NA, data were not available; NS, no statistical significance.

a Fisher's exact test with *p* < .05 considered significant*.

Renal anomalies in HDR syndrome have been widely described, including congenital renal aplasia, hypoplasia, or dysplasia (41%), vesico‐ureteral reflux (16%), and cysts or pelvicalyceal deformities (11%) (Belge et al., 2016). Our patient presented a right renal cyst, which is uncommon in HDR patients. Therefore, the co‐occurrence of left renal aplasia and hypercalciuria is deemed to advance the appearance of renal failure and increase the complexity of kidney disease in the 6‐year‐old boy.

Studies have documented the increased risks of enamel hypomineralization in patients with hypocalcemia (Hejlesen et al., 2018; Kelly, Pomarico, & Souza, 2009; Nikiforuk & Fraser, 1981). Moreover, enamel hypomineralization has been reported in relation to the diseases with hypoparathyroidism (which can result in hypocalcemia), such as 22q11 deletion syndrome (Hejlesen et al., 2018; Nordgarden et al., 2012). However, it has been rarely described in the HDR syndrome studies, and the known teeth finding was fluorosis caused by excessive fluoride intake (Chen et al., 2015). In this study, the proband showed enamel hypomineralization of the permanent first molars and incisors and hypocalcemia as the result of hypoparathyroidism. In addition, his new permanent molars were normal after calcium and calcitriol supplementation. Therefore, we hypothesized that enamel hypomineralization is the outcome of hypoparathyroidism, and it may be overlooked without pertinent examination, or may remain undetected if not evaluated at the appropriate age (dental amelogenesis stage).

In conclusion, we describe a patient with the triad of HDR, enamel hypomineralization, and early‐onset hypercalciuria, which is atypical for HDR syndrome. The presence of the triad of HDR and enamel hypomineralization may be caused by the frameshift variants in *GATA3*; the atypical hypercalciuria occurring both in the patient and his father may be explained by the heterozygous missense variant in *SLC34A3*. Furthermore, this report reaffirmed the importance of the trio whole‐exome sequencing and consideration of other genetic causes for patients with complex clinical phenotypes.

## ETHICS STATEMENT

Informed consent was obtained from the family.

## CONFLICT OF INTEREST

The authors declare no conflict of interests.

## AUTHOR CONTRIBUTIONS

Z‐mX and SY designed the study. HW supervised the work and performed the genetic analysis. WL performed endocrine functional analysis. Other co‐authors provided patient care and collected the data. SY and HW drafted the manuscript. All authors reviewed, edited, and approved the final manuscript.

## Supporting information

Table S1

Table S2

Appendix S1

## Data Availability

The data that support the findings of this study are available upon request from the corresponding author. The data are not publicly available due to privacy or ethical restrictions.
