# Supplementary material for: Novel heterozygous GATA3 and SLC34A3 variants in a 6‐year‐old boy with Barakat syndrome and hypercalciuria
Source: Mol Genet Genomic Med. 2020 Mar 10;8(5):e1222. doi: 10.1002/mgg3.1222 (PMC7216807; doi:10.1002/mgg3.1222)
Supplement: Supplementary file 1 — Table S1 [file MGG3-8-e1222-s001.docx]

**TABLE S1.** Reported patients with *GATA3* mutation and their audiological characteristics

| **Patient** | **Gender** | **Genome variant** | **Mutation type** | **Age at diagnose of HL** | **Characteristics of HL** | | | | **Configuration of HL** | **PTA**  **(dB HL,left)** | **PTA**  **(dB HL, right)** | **ABR**  **(dB HL, left)** | **ABR**  **(dB HL, right)** |
| --- | --- | --- | --- | --- | --- | --- | --- | --- | --- | --- | --- | --- | --- |
|  |  |  |  |  | **Type**  **Bilateral** **Symmetrical Progressive** | | | |  |  |  |  |  |
| # 1^1^ | female | c.784A>G (p. R262G) | missense | 11 y | SNHL | bilateral | NA | NA | NA | 80-100 | 60 | NA | NA |
| # 2^1^ | male | c.942T>A (p. C318S) | missense | 8 y | SNHL | bilateral | NA | NA | NA | 40-50 | 40-50 | NA | NA |
| # 3^2^ | male | c.896G>A (p. R299Q) | missense | 1y | SNHL | bilateral | asymmetrical | NA | High-frequency HL | 50 | 60 | NA | NA |
| # 4^3^ | male | c.815C>T (p. T272I) | missense | 8 m | SNHL | bilateral | NA | NA | NA | 60 | 60 | NA | NA |
| # 5^4^ | male | c.823T>A (p. W275R) | missense | childhood | SNHL | bilateral | symmetrical | NA | NA | 70 | 70 | NA | NA |
| # 6^5^ | female | c.824G>T (p. W275L) | missense | 11 y | SNHL | bilateral | NA | NA | NA | 60 | 60 | NA | NA |
| # 7^6^ | female | c.896G>A (p. R299Q) | missense | 47 y | SNHL | bilateral | NA | progressive | NA | 55 | 47 | NA | NA |
| # 8^6^ | female | c.896G>A (p. R299Q) | missense | 14 y | SNHL | bilateral | NA | NA | NA | 47 | 56 | NA | NA |
| # 9^1^ | female | c.1051-1G>T  (p.I351fsX18) | splice | 5 m | SNHL | bilateral | NA | NA | NA | 80 | 80 | NA | NA |
| # 10^2^ | female | c.924+4_924+19del | splice | Infancy | SNHL | bilateral | NA | NA | NA | >50 | 50 | NA | NA |
| # 11^2^ | female | c.1051-2A>G | splice | 1 y | SNHL | bilateral | NA | NA | NA | 70 | 70 | NA | NA |
| # 12^5^ | male | c.1050+1G>C | splice | 12 m | SNHL | bilateral | NA | NA | NA | 50 | 50 | NA | NA |
| # 13^7^ | male | c.924+5G>C  (p.Glu260ValfsX43) | splice | 9 y | SNHL | bilateral | NA | NA | NA | 45 | 45 | NA | NA |
| # 14^8^ | male | c.1099C > T (p.Arg367*) | nonsense | < 11 m | SNHL | bilateral | symmetrical | non-progressive | Flat | 55 | 56.67 | NA | NA |
| # 15^8^ | female | c.1099C > T (p.Arg367*) | nonsense | < 4 y | SNHL | bilateral | symmetrical | non-progressive | flat | 55 | 56.7 | NA | NA |
| # 16^9^ | male | c.465-513 deletion | deletion | 8 y | SNHL | bilateral | symmetrical | non-progressive | Gently sloping | 55 | 51.6 | NA | NA |
| # 17^5^ | male | 10Mb deletion | deletion | 6 m | SNHL | bilateral | NA | NA | NA | 90 | 90 | NA | NA |
| # 18^10^ | male | 419025Mb deletion | deletion | 4 y | SNHL | bilateral | NA | NA | NA | 110 | 110 | NA | NA |
| # 19^11^ | male | 1.5Mb deletion | deletion | 6 m | SNHL | bilateral | NA | NA | NA | NA | NA | >90 | >90 |
| # 20^12^ | female | GATA3 gene & 7 others | deletion | NA | SNHL | bilateral | NA | NA | NA | NA | NA | 85 | 50 |
| # 21^12^ | female | 10p13/14 | deletion | NA | SNHL | bilateral | NA | NA | NA | NA | NA | 70 | 70 |
| # 22^12^ | male | 10p13/14 | deletion | NA | SNHL | bilateral | NA | NA | NA | NA | NA | 85 | 85 |
| # 23^13^ | male | del (10)(p13) | deletion | 2 y | SNHL | bilateral | symmetrica | Non-progressive | NA | NA | NA | 90-100 | 90-100 |
| # 24^14^ | male | 10p14-p15.1. | deletion | 7 y | SNHL | bilateral | NA | NA | NA | NA | NA | 40-50 | 40-50 |
| # 25^15^ | male | 900-kb deletion | deletion | 1 y | SNHL | bilateral | symmetrical | non-progressive | Steeply sloping | 60 | 53.33 | NA | NA |
| # 26^16^ | female | 250-kb deletion | deletion | 8 y | SNHL | bilateral | NA | progressive | NA | 50 | 50 | NA | NA |
| # 27^1^ | female | c.1063delC(p.L355*) | frameshift | 2.8 y | SNHL | bilateral | NA | NA | NA | 60 | 60 | NA | NA |
| # 28^1^ | female | c.432insG(p.K303*) | frameshift | 8 y | SNHL | bilateral | NA | NA | NA | 45 | 60 | NA | NA |
| # 29^17^ | female | c.149delT (p.Phe51LeufsX144) | frameshift | 16 y | SNHL | bilateral | NA | NA | Flat | 66.7 | 66.7 | NA | NA |
| # 30^17^ | female | c.149delT (p.Phe51LeufsX144) | frameshift | 16 y | SNHL | bilateral | NA | NA | Flat | 63.33 | 63.33 | NA | NA |
| # 31^17^ | female | c.149delT (p.Phe51LeufsX144) | frameshift | 16 y | SNHL | bilateral | NA | NA | Flat | 60 | 60 | NA | NA |
| # 32^17^ | female | c.149delT (p.Phe51LeufsX144) | frameshift | 7 y | SNHL | bilateral | NA | NA | Flat | 45 | 45 | NA | NA |
| # 33^18^ | male | c.523_528dup (p.Gln178ProfsX19) | frameshift | 1.6 y | SNHL | bilateral | symmetrical | non-progressive | Flat | 55 | 55 | NA | NA |
| # 34^18^ | female | c.523_528dup (p.Gln178ProfsX19) | frameshift | 1.6 y | SNHL | bilateral | symmetrical | non-progressive | Flat | 43.33 | 43.33 | NA | NA |
| # 35^18^ | female | c.523_528dup (p.Gln178ProfsX19) | frameshift | 2.89 y | SNHL | bilateral | symmetrical | non-progressive | Flat | 55 | 55 | NA | NA |
| # 36^18^ | female | c.523_528dup (p.Gln178ProfsX19) | frameshift | 5.29 y | SNHL | bilateral | symmetrical | non-progressive | Flat | 41.67 | 41.67 | NA | NA |
| # 37^18^ | male | c.523_528dup (p.Gln178ProfsX19) | frameshift | 6.65 y | SNHL | bilateral | symmetrical | non-progressive | Flat | 31.67 | 31.67 | NA | NA |
| # 38^9^ | female | c.404dupC p.(Ala136Glyfs*168) | frameshift | 4 y | SNHL | bilateral | symmetrical | progressive | Gently sloping | 58.3 | 56.67 | 90 | 90 |
| # 39^19^ | female | c.708_709insC(p.Ser237Glnfs*66) | frameshift | 6 y | SNHL | bilateral | symmetrical | NA | flat | 36.67 | 33.33 | NA | NA |
| # 40^19^ | male | c.708_709insC(p.Ser237Glnfs*66) | frameshift | 5 y | SNHL | bilateral | symmetrical | NA | Gently sloping | 50 | 45 | NA | NA |
| # 41^19^ | female | c.708_709insC(p.Ser237Glnfs*66) | frameshift | 6 y | SNHL | bilateral | symmetrical | progressive | flat | 41.67 | 45 | NA | NA |
| # 42^19^ | male | c.708_709insC(p.Ser237Glnfs*66) | frameshift | 50 y | SNHL | bilateral | symmetrical | NA | Gently sloping | 50 | 55 | NA | NA |
| # 43^4^ | female | c.901delCinsAACCCT p.(Leu301Asnfs*57) | frameshift | 27 y | SNHL | bilateral | symmetrical | NA | NA | 40-50 | 40-50 | NA | NA |
| # 44^4^ | male | c.901delCinsAACCCT (p.Leu301Asnfs*57) | frameshift | childhoood | SNHL | bilateral | symmetrical | NA | NA | >110 | 60-70 | NA | NA |
| # 45^4^ | female | c.404dupC p.(Ala136Glyfs*168) | frameshift | 13 y | SNHL | bilateral | symmetrical | NA | NA | 50 | 50 | NA | NA |
| # 46^4^ | female | c.700_701delTTinsCTC p.(Phe234Leufs*70) | frameshift | 6 y | SNHL | bilateral | symmetrical | NA | NA | >70 | >70 | NA | NA |
| # 47^20^ | male | c.35_36delGC(p.Ser12ThrfsSTOP40) | frameshift | 4 y | SNHL | bilateral | symmetrical | NA | flat | 48.33 | 51.67 | NA | NA |
| # 48^21^ | female | c.1200_1201delca; p.h400fsX506 | frameshift | infancy | SNHL | bilateral | NA | NA | NA | NA | NA | 80 | 80 |
| # 49^22^ | female | c.1200_1201delca; p.h400fsX506 | frameshift | 12 m | SNHL | bilateral | NA | NA | NA | NA | NA | 100 | 80 |
| # 50^23^ | male | c.252_255dupGTGC ( p.Arg86Valfs*219) | frameshift | 12 y | SNHL | bilateral | symmetrical | NA | Gently sloping | 38.33 | 31.67 | NA | NA |
| # 51^24^ | female | c.431delG p.(Gly144Alafs*51) | frameshift | 8 y | SNHL | bilateral | symmetrical | non-progressive | Gently sloping | 43.33 | 43.33 | NA | NA |
| # 52^24^ | female | c.431delG p.(Gly144Alafs*51) | frameshift | 6 y | SNHL | bilateral | symmetrical | non-progressive | flat | 76.67 | 76.67 | NA | NA |
| # 53^25^ | male | c. 529dupC (p. Arg177profs*126) | frameshift | 2 y | M | bilateral | symmetrical | non-progressive | rising | 90 | 86.67 | NA | NA |

ABR, auditory brainstem response; HL, hearing loss; M, mixed hearing loss; PTA, pure tone audiometry; NA, data was not available

**Reference**

1. Nakamura A, Fujiwara F, Hasegawa Y, et al. Molecular analysis of the GATA3 gene in five Japanese patients with HDR syndrome. *Endocr J*. 2011;58(2):123-130. doi:10.1507/endocrj.K10E-246

2. Belge H, Dahan K, Cambier J-F, et al. Clinical and mutational spectrum of hypoparathyroidism, deafness and renal dysplasia syndrome. *Nephrol Dial Transplant*. July 2016:gfw271. doi:10.1093/ndt/gfw271

3. Gomes T, Gortner L, Dockter G, Leitner D, Thakker R, Rohrer T. HDR Syndrome – A Follow-up Genotype-Phenotype Analysis of a de novo Missense Thr272Ile Mutation in Exon 4 of GATA3. *Klin Padiatr*. 2012;224(07):452-454. doi:10.1055/s-0032-1329947

4. Muroya K, Hasegawa T, Ito Y, et al. GATA3 abnormalities and the phenotypic spectrum of HDR syndrome. *J Med Genet*. 2001;38(6):374-380. doi:10.1136/jmg.38.6.374

5. Fukami M, Muroya K, Miyake T, et al. GATA3 abnormalities in six patients with HDR syndrome. *Endocr J*. 2011;58(2):117-121. doi:10.1507/endocrj.K10E-234

6. Okawa T, Yoshida M, Usui T, et al. A novel loss-of-function mutation of GATA3 (p.R299Q) in a Japanese family with Hypoparathyroidism, Deafness, and Renal Dysplasia (HDR) syndrome. *BMC Endocr Disord*. 2015;15(1):66. doi:10.1186/s12902-015-0065-7

7. Matsuo K, Kobayashi A, Tanahashi Y, et al. Novel splice site mutation in *GATA3* in a patient with HDR syndrome. *Clin Pediatr Endocrinol*. 2017;26(4):271-273. doi:10.1297/cpe.26.271

8. Martins FTA, Ramos BD, Sartorato EL. A rare case of deafness and renal abnormalities in HDR syndrome caused by a de novo mutation in the GATA3 gene. *Genet Mol Biol*. 2018;41(4):794-798. doi:10.1590/1678-4685-gmb-2017-0194

9. van Looij MAJ, Meijers-Heijboer H, Beetz R, et al. Characteristics of Hearing Loss in HDR (Hypoparathyroidism, Sensorineural Deafness, Renal Dysplasia) Syndrome. *Audiol Neurotol*. 2006;11(6):373-379. doi:10.1159/000095899

10. Melis D, Genesio R, Boemio P, et al. Clinical description of a patient carrying the smallest reported deletion involving 10p14 region. *Am J Med Genet*. 2012;158A(4):832-835. doi:10.1002/ajmg.a.34133

11. Fukai R, Ochi N, Murakami A, et al. Co-occurrence of 22q11 deletion syndrome and hdr syndrome. *Am J Med Genet*. July 2013:n/a-n/a. doi:10.1002/ajmg.a.36083

12. Lichtner P, König R, Hasegawa T, Van Esch H, Meitinger T, Schuffenhauer S. An HDR (hypoparathyroidism, deafness, renal dysplasia) syndrome locus maps distal to the DiGeorge syndrome region on 10p13/14. *J Med Genet*. 2000;37(1):33-37. doi:10.1136/jmg.37.1.33

13. Hasegawa T, Hasegawa Y, Aso T, et al. HDR syndrome (hypoparathyroidism, sensorineural deafness, renal dysplasia) associated with del(10)(p13). *Am J Med Genet*. 1997;73(4):416-418. doi:10.1002/(sici)1096-8628(19971231)73:4<416::aid-ajmg9>3.0.co;2-l

14. Fujimoto S, Yokochi K, Morikawa H, et al. Recurrent cerebral infarctions and del(10)(p14p15.1) de novo in HDR (hypoparathyroidism, sensorineural deafness, renal dysplasia) syndrome. *Am J Med Genet*. 1999;86(5):427-429. doi:10.1002/(sici)1096-8628(19991029)86:5<427::aid-ajmg6>3.0.co;2-i

15. Bilous RW, Murty G, Parkinson DB, et al. Brief report: autosomal dominant familial hypoparathyroidism, sensorineural deafness, and renal dysplasia. *N Engl J Med*. 1992;327(15):1069-1074. doi:10.1056/NEJM199210083271506

16. Esch HV, Groenen P, Daw S, et al. Partial DiGeorge syndrome in two patients with a 10p rearrangement. *Clinical Genetics*. 1999;55(4):269-276. doi:10.1034/j.1399-0004.1999.550410.x

17. Lin Y-H, Wu C-C, Hsu T-Y, Chiu W-Y, Hsu C-J, Chen P-L. Identification of a novel GATA3 mutation in a deaf Taiwanese family by massively parallel sequencing. *Mutation Research/Fundamental and Molecular Mechanisms of Mutagenesis*. 2015;771:1-5. doi:10.1016/j.mrfmmm.2014.11.001

18. van Beelen E, Leijendeckers JM, Admiraal RJC, et al. Audiometric Characteristics of a Dutch Family with a New Mutation in  ***GATA3***  Causing HDR Syndrome. *Audiol Neurotol*. 2014;19(2):106-114. doi:10.1159/000356303

19. Kita M, Kuwata Y, Usui T. Familial congenital choanal atresia with GATA3 associated hypoparathyroidism-deafness-renal dysplasia syndrome unidentified on auditory brainstem response. *Auris Nasus Larynx*. 2019;46(5):808-812. doi:10.1016/j.anl.2018.10.005

20. Al-Shibli A, Al Attrach I, Willems PJ. Novel DNA mutation in the GATA3 gene in an Emirati boy with HDR syndrome and hypomagnesemia. *Pediatr Nephrol*. 2011;26(7):1167-1170. doi:10.1007/s00467-011-1835-8

21. Cheon C-K, Kim G-H, Yoo H-W. The First Korean Case of HDR Syndrome Confirmed by Clinical and Molecular Investigation. *Yonsei Med J*. 2015;56(1):300. doi:10.3349/ymj.2015.56.1.300

22. Muroya K, Mochizuki T, Fukami M, et al. Diabetes Mellitus in a Japanese Girl with HDR Syndrome and GATA3 Mutation. *Endocr J*. 2010;57(2):171-174. doi:10.1507/endocrj.K09E-313

23. Shim YS, Choi W, Hwang IT, Yang S. Hypoparathyroidism, sensorineural deafness, and renal dysgenesis syndrome with a GATA3 mutation. *Ann Pediatr Endocrinol Metab*. 2015;20(1):59-63. doi:10.6065/apem.2015.20.1.59

24. Hernández AM, Villamar M, Roselló L, Moreno‐Pelayo MA, Moreno F, Castillo I del. Novel mutation in the gene encoding the GATA3 transcription factor in a Spanish familial case of hypoparathyroidism, deafness, and renal dysplasia (HDR) syndrome with female genital tract malformations. *American Journal of Medical Genetics Part A*. 2007;143A(7):757-762. doi:10.1002/ajmg.a.31617

25. Chen L, Chen B, Leng W, et al. Identification of a novel de novo GATA3 mutation in a patient with HDR syndrome. *J Int Med Res*. 2015;43(5):718-724. doi:10.1177/0300060515591065
