# Supplementary material for: Novel heterozygous GATA3 and SLC34A3 variants in a 6‐year‐old boy with Barakat syndrome and hypercalciuria
Source: Mol Genet Genomic Med. 2020 Mar 10;8(5):e1222. doi: 10.1002/mgg3.1222 (PMC7216807; doi:10.1002/mgg3.1222)
Supplement: Supplementary file 2 — Table S2 [file MGG3-8-e1222-s002.docx]

**Table S2.** Degree of hearing loss according to WHO

| **Degree of hearing loss** | **Hearing threshold (dB)** |
| --- | --- |
| Normal | ≤25 |
| Mild | 26-40 |
| Moderate | 41-55 |
| Moderately severe | 56-70 |
| Severe | 71-90 |
| profound | 90 |
