# Supplementary material for: Novel heterozygous GATA3 and SLC34A3 variants in a 6‐year‐old boy with Barakat syndrome and hypercalciuria
Source: Mol Genet Genomic Med. 2020 Mar 10;8(5):e1222. doi: 10.1002/mgg3.1222 (PMC7216807; doi:10.1002/mgg3.1222)
Supplement: Supplementary file 3 — Appendix S1 [file MGG3-8-e1222-s003.docx]

## Audiological classifications

Sensorineural hearing loss (SNHL) was defined with an air-bone gap of less than 15 dB average over 0.5, 1 and 2 kHz in addition to a pure tone average of 0.5, 1, 2 and 4 kHz (PTA4) of more than 20 dB HL. Conductive HL was defined as normal bone conduction thresholds but an air-bone gap over 15 dB in the average at 0.5, 1 and 2 kHz. Mixed HL was determined if the bone conduction threshold was greater than 20 dB HL with an air-bone gap of more than 15 dB in average of 0.5, 1 and 2 kHz.

The degree of SNHL was classified by the pure tone average at 0.5, 1, and 2 kHz of the better ear according to the classifications shown in Table S2.

Audiogram configurations of HL were sorted according to the modified hearing impairment classification by Hederstierna et al and subdivided into five different types: rising, mid-frequency u-shaped, high-frequency u-shaped, high-frequency sloping (gently and steeply) and flat.^1^ Moreover, the audiograms that did not meet any of the criteria above are classified as unspecified.

## Genetic studies

Genomic DNA was extracted from whole blood using the QIAamp DNA Blood Mini Kit (QIAGEN, Germany) according to the manufacturer’s protocol. DNA fragments were enriched for exome sequences using the Agilent SureSelect XT Human All Exon 50 Mb kit.

Sequence data were mapped to the human reference genome (GRCh37/hg19). Variants were annotated by ANNOVAR and VEP software^2^, with a minor allele frequency of less than 5% according to either the 1,000 Genomes Project or the Exome Aggregation Consortium (ExAC). Missense variants were evaluated by the SIFT, PolyPhen2.2 and MutationTaster.^3,4,5^

The pathogenicity of the candidate variants was analyzed according to the standards and guidelines recommended by the American College of Medical Genetics and Genomics (ACMG) and only pathogenic variants were enrolled in the present study. The candidate variants were validated by Sanger sequencing on an ABI 3730 Genetic Analyzer (Applied Biosystems, Foster City, CA, USA), and the same variant was detected in their parents to confirm whether the variant was germline-derived.

## Reference

1. Hederstierna C, Hultcrantz M, Collins A, Rosenhall U. Hearing in women at menopause. Prevalence of hearing loss, audiometric configuration and relation to hormone replacement therapy. *Acta Otolaryngol*. 2007;127(2):149-155. doi:10.1080/00016480600794446

2. McLaren W, Pritchard B, Rios D, Chen Y, Flicek P, Cunningham F. Deriving the consequences of genomic variants with the Ensembl API and SNP Effect Predictor. *Bioinformatics*. 2010;26(16):2069-2070. doi:10.1093/bioinformatics/btq330

3. Kumar P, Henikoff S, Ng PC. Predicting the effects of coding non-synonymous variants on protein function using the SIFT algorithm. *Nat Protoc*. 2009;4(7):1073-1081. doi:10.1038/nprot.2009.86

4. Adzhubei IA, Schmidt S, Peshkin L, et al. A method and server for predicting damaging missense mutations. *Nat Methods*. 2010;7(4):248-249. doi:10.1038/nmeth0410-248

5. Schwarz JM, Rödelsperger C, Schuelke M, Seelow D. MutationTaster evaluates disease-causing potential of sequence alterations. *Nat Methods*. 2010;7(8):575-576. doi:10.1038/nmeth0810-575
